# Absence of Desmin Results in Impaired Adaptive Response to Mechanical Overloading of Skeletal Muscle

**DOI:** 10.3389/fcell.2021.662133

**Published:** 2021-07-15

**Authors:** Pierre Joanne, Yeranuhi Hovhannisyan, Maximilien Bencze, Marie-Thérèse Daher, Ara Parlakian, Geraldine Toutirais, Jacqueline Gao-Li, Alain Lilienbaum, Zhenlin Li, Ekaterini Kordeli, Arnaud Ferry, Onnik Agbulut

**Affiliations:** ^1^Sorbonne Université, Institut de Biologie Paris-Seine (IBPS), CNRS UMR 8256, Inserm ERL U1164, Biological Adaptation and Ageing, Paris, France; ^2^U955-IMRB, Team 10, Biology of the Neuromuscular System, Inserm, UPEC, ENVA, EFS, Créteil, France; ^3^Muséum National d’Histoire Naturelle (MNHN), Unité Molécules de Communication et Adaptation des Micro-organismes (MCAM), CNRS UMR 7245, Plateau technique de Microscopie Electronique (PtME), Paris, France; ^4^Unité de Biologie Fonctionnelle et Adaptative, CNRS UMR 8251, Université de Paris, Paris, France; ^5^Institut de Myologie, INSERM U974, Centre de Recherche en Myologie, Sorbonne Université, Paris, France; ^6^Université de Paris, Paris, France

**Keywords:** intermediate filament, cytoskeleton, muscle hypertrophy, autophagy, exercice

## Abstract

**Background:** Desmin is a muscle-specific protein belonging to the intermediate filament family. Desmin mutations are linked to skeletal muscle defects, including inherited myopathies with severe clinical manifestations. The aim of this study was to examine the role of desmin in skeletal muscle remodeling and performance gain induced by muscle mechanical overloading which mimics resistance training.

**Methods:** Plantaris muscles were overloaded by surgical ablation of gastrocnemius and soleus muscles. The functional response of plantaris muscle to mechanical overloading in desmin-deficient mice (*Des*KO, *n* = 32) was compared to that of control mice (*n* = 36) after 7-days or 1-month overloading. To elucidate the molecular mechanisms implicated in the observed partial adaptive response of *Des*KO muscle, we examined the expression levels of genes involved in muscle growth, myogenesis, inflammation and oxidative energetic metabolism. Moreover, ultrastructure and the proteolysis pathway were explored.

**Results:** Contrary to control, absolute maximal force did not increase in *Des*KO muscle following 1-month mechanical overloading. Fatigue resistance was also less increased in *Des*KO as compared to control muscle. Despite impaired functional adaptive response of *Des*KO mice to mechanical overloading, muscle weight and the number of oxidative MHC2a-positive fibers per cross-section similarly increased in both genotypes after 1-month overloading. However, mechanical overloading-elicited remodeling failed to activate a normal myogenic program after 7-days overloading, resulting in proportionally reduced activation and differentiation of muscle stem cells. Ultrastructural analysis of the plantaris muscle after 1-month overloading revealed muscle fiber damage in *Des*KO, as indicated by the loss of sarcomere integrity and mitochondrial abnormalities. Moreover, the observed accumulation of autophagosomes and lysosomes in *Des*KO muscle fibers could indicate a blockage of autophagy. To address this issue, two main proteolysis pathways, the ubiquitin-proteasome system and autophagy, were explored in *Des*KO and control muscle. Our results suggested an alteration of proteolysis pathways in *Des*KO muscle in response to mechanical overloading.

**Conclusion:** Taken together, our results show that mechanical overloading increases the negative impact of the lack of desmin on myofibril organization and mitochondria. Furthermore, our results suggest that under these conditions, the repairing activity of autophagy is disturbed. Consequently, force generation is not improved despite muscle growth, suggesting that desmin is required for a complete response to resistance training in skeletal muscle.

## Introduction

Desmin belongs to the family of intermediate filaments and is specifically expressed in skeletal, smooth and cardiac muscle cells. In absence of desmin, or due to mutations in the encoding gene, several defects have been described in all three muscle types, and particularly in cardiac (Brodehl et al., 2018) and skeletal muscle (Goldfarb and Dalakas, 2009; van Spaendonck-Zwarts et al., 2010). In skeletal muscle cells, desmin forms filaments that connect different organelles between them, to the cytoskeleton, and to the plasma membrane. Desmin filaments are linked to the costameres and Z-discs through interactions with synemin (Granger and Lazarides, 1980; Bellin et al., 2001), plectin (Konieczny et al., 2008), nebulette and indirectly to actin filaments (Hernandez et al., 2016), contributing to its role in the maintenance of the structural and mechanical integrity of the contractile apparatus in muscle tissues.

Since their generation (Li et al., 1996; Milner et al., 1996), the desmin knock-out mice (*Des*KO) were used to assess the role of this intermediate filament in skeletal muscles. Under resting conditions, desmin-deficient soleus muscle shows an increase in the number of slow/oxidative fibers, but also a decrease in force and fatigue resistance, as compared to control (Li et al., 1997). However, the role of desmin in the long-term adaptation process following extreme muscle mechanical stimulation is not clear. It has been suggested that desmin is protective in the case of remodeling induced by endurance exercice and, on the contrary, deleterious when the muscle is submitted to eccentric exercise (Sam et al., 2000).

Mechanical overloading (OVL) of plantaris muscle mimics resistance training and powerfully induces muscle remodeling and hypertrophy as well as increase in muscle function. In response to OVL, the weight of plantaris muscle almost doubles, absolute maximal force and fatigue resistance increase, and muscle fibers exhibit a fast/glycolytic to slow/oxidative type transition (Ianuzzo et al., 1976; Roy and Edgerton, 1995; Parsons et al., 2004; Joanne et al., 2012). Since desmin expression can be increased in response to resistance training (Woolstenhulme et al., 2006; Parcell et al., 2009), in this study, we addressed the putative role of desmin on muscle remodeling and performance gain induced by OVL in mice.

Herein, we compared the effects of 1 month-OVL on absolute maximal force, specific maximal force, fatigue resistance, muscle growth, and fiber type transition of the plantaris muscle of adult desmin-deficient mice (*Des*KO) and of age- and sex-matched control mice. We collected evidence of a dichotomy between the OVL- induced muscle gain of mass, and the effects on muscle function in *DesKO* muscles compared to controls. Our data suggest that the down regulation of the myostatin pathway efficiently promotes muscle hypertrophy in *DesKO* muscles. However, OVL-elicited remodeling failed to activate normal myogenic program, resulting in proportionally less muscle stem cell (MuSC) activation and differentiation. Furthermore, desmin absence prevented the upregulation of LC3, suggesting that there is a link between desmin and the autophagic process that accompanies muscle remodeling following mechanical overloading.

## Materials and Methods

### Animals and Treatments

All procedures were performed in accordance with national and European legislations, in conformity with the Public Health Service Policy on Human Care and Use of Laboratory Animals under the license 75-1102. 32 2-months-old *Des*KO female mice were used in this study. Age-matched wild-type (*n* = 24) or *Des*^+/–^ heterezygous (*n* = 12) female mice were used as controls. Mice were randomly divided into different control and experimental groups. All animal studies were approved by our institutional Ethics Committee (Charles Darwin, project number: 01362.02) and conducted according to the French and European laws, directives, and regulations on animal care (European Commission Directive 86/609/EEC). Our animal facility is fully licensed by the French competent authorities and has animal welfare insurance. For OVL, the mice were anesthetized with pentobarbital (50 mg/kg body weight, i.p.). The plantaris muscles of both legs were mechanically overloaded for 7 days or 1 month by the surgical removal of soleus muscles and a major portion of the gastrocnemius muscles as described (Joanne et al., 2012; Ferry et al., 2015).

### Muscle Force Measurements

Plantaris muscle function was evaluated by measuring *in situ* isometric force, as described (Vignaud et al., 2007; Hourdé et al., 2013a). Briefly, mice were anesthetized (pentobarbital sodium, 50 mg/kg, i.p.). During physiological experiments, supplemental doses were given as required, to maintain deep anesthesia. The knee and foot were fixed with clamps and stainless-steel pins. The plantaris muscle was exposed (and the distal tendon of the gastrocnemius and soleus muscle complex was cut in non-overloaded muscles). The distal tendon of the plantaris muscle was attached to an isometric transducer (Harvard Apparatus) with a silk ligature. The sciatic nerves were proximally crushed and distally stimulated by a bipolar silver electrode using supramaximal square wave pulses of 0.1 ms duration. Responses to tetanic stimulation (pulse frequency 75–143 Hz) were successively recorded. At least 1 min was allowed between contractions. Absolute maximal forces were determined at optimal length (length at which maximal tension was obtained during the tetanus). Force was normalized to the muscle mass (m) as an estimate of specific maximal force. Fatigue resistance was then determined after a 5-min rest period. The muscle was continuously stimulated at 50 Hz for 2 min (submaximal continuous tetanus). The duration corresponding to a 50% decrease in force was noted. Body temperature was maintained at 37°C using radiant heat. After the measurements, mice were euthanized with an overdose of pentobarbital.

### Histology and Immunohistochemistry

Transverse 10 μm-thick frozen sections were prepared from the mid-belly region of plantaris muscles using a cryostat (Leica Microsystems, Nanterre, France). Some sections were processed for histological analysis (Hematoxylin-eosin, Sirius red stainings) according to standard protocols. Other sections were processed for immunohistochemistry as described previously (Joanne et al., 2012; Hourdé et al., 2013b). Briefly, the sections were incubated with primary antibodies against heparan sulfate proteoglycan (Perlecan) (1:400, rat monoclonal, Millipore), myosin heavy chain (MHC)-2a (1:50, mouse monoclonal, clone SC-71, Developmental Studies Hybridoma Bank, University of Iowa) or MHC-2b (1:5, mouse monoclonal, clone BF-F3, Developmental Studies Hybridoma Bank, University of Iowa). After washing in PBS, sections were incubated 1 h with secondary antibodies (Alexa Fluor^®^, Life Technologies). After washing in PBS, slides were finally mounted using mowiol containing 5 μg/ml Hoescht 33342 (Life Technologies). Images were captured using a motorized fluorescent microscope (Dmi8, Leica Microsystems). Morphometric analyses were made using the ImageJ software and a custom macro as described previously (Joanne et al., 2012; Hourdé et al., 2013a). The percentage of fiber type and smallest diameter (min-Ferret) of all muscle fibers of the whole muscle section were measured.

### Electron Microscopy

Electron microscopy was carried out as described previously (Agbulut et al., 2001; Joanne et al., 2013). Briefly, the calf muscles of mice were fixed in 2% glutaraldehyde and 2% paraformaldéhyde in 0.2 M phosphate buffer at pH 7.4 for 1 h at room temperature. After 1 h, the plantaris muscle was dissected and separated in three by a short-axis section, then fixed overnight at 4°C in the same fixative. After washing, specimens were post-fixed for 1 h with 1% osmium tetroxide solution, dehydrated in increasing concentrations of ethanol and finally in acetone, and embedded in epoxy resin. The resin was polymerized for 48 h at 60°C. Ultrathin sections (70 nm) were cut with an ultramicrotome (Leica UC6, Leica Microsystems), picked-up on copper rhodium-coated grids and stained for 2 min with Uranyl-Less solution (Delta Microscopies, France) and 2 min with 0.2% lead citrate before observation at 80 kV with an electron microscope (912 Omega, Zeiss) equipped with a digital camera (Veleta 2kx2k, Emsis, Germany).

### Western-Blotting

Immunoblotting was carried out as described previously (Joanne et al., 2012; Li et al., 2014). Muscle tissues were snap-frozen in liquid nitrogen immediately after dissection. Frozen muscles were placed into an ice-cold homogenization buffer containing: 50 mM Tris (pH 7.6), 250 mM NaCl, 3 mM EDTA, 3 mM EGTA, 0.5% NP40, 2 mM dithiothreitol, 10 mM sodium orthovanadate, 10 mM NaF, 10 mM glycerophosphate and 2% of protease inhibitor cocktail (Sigma-Aldrich). Samples were minced with scissors and homogenized using plastic pestles, incubated 30 min on ice, sonicated 3 times for 5 s with 30-s intervals on ice, then centrifuged at 12,000 g for 30 min at 4°C. Protein concentration was measured using the Bradford method with bovine serum albumin as a standard. Equal amounts of protein extracts (25 μg) were separated by SDS-PAGE before electrophoretic transfer onto a nitrocellulose membrane (GE Healthcare). Western-blot analysis was carried out using anti-total-protein kinase A regulatory subunit IIα (PKARIIα) (1:1,00, rabbit polyclonal, Millipore), anti-phospho-PKARIIα (Ser96) (1:1,000, rabbit polyclonal, Millipore), anti-LC3-II (1:1,000, rabbit polyclonal, Sigma-Aldrich), anti-β-tubulin (1:1,000, mouse monoclonal, Sigma-Aldrich) and anti-Glyceraldehyde 3-phosphate dehydrogenase (GAPDH) antibody (1:5,000, mouse monoclonal, Santa Cruz Biotechnology). Proteins bound to primary antibodies were visualized with peroxidase-conjugated secondary antibodies (Thermo Fisher Scientific) and a chemiluminescent detection system (ECL-Plus, GE Healthcare). Bands were quantified by densitometric software (Multi Gauge, Fujifilm). At least three animals were used for each experimental point. The levels of activation of autophagy were calculated by quantification of the LC3-II (anti-LC3 antibody, Sigma-Aldrich) band normalized to β-tubulin (anti-β-tubulin antibody) (Sarkar et al., 2009).

### Proteasome Activity Measurement

Tissue homogenates containing proteasome were prepared just after dissection using ice-cold homogenization buffer containing: 20 mM Tris-HCl (pH 7.6), 250 mM NaCl, 3 mM EDTA, 3 mM EGTA and 2 mM DTT (Hovhannisyan et al., 2019). Samples were minced with scissors and homogenized using plastic pestles, incubated 30 min on ice, then centrifuged at 12,000 g for 15 min at 4°C. Protein concentration was measured using the Bradford method with bovine serum albumin as a standard. The proteasomal chymotrypsin-like, trypsin-like and caspase-like activities of the 20S catalytic core were assayed using the fluorogenic substrates *N*-Succinyl-Leu-Leu-Val-Tyr-7-amino-4-methylcoumarin (Suc-LLVY-AMC, Enzo Life Sciences), Bz-Val-Gly-7-amino-4-methylcoumarin (Bz-VGR-AMC, Enzo Life Sciences) and Z-Leu-Leu-Glu-7-amino-4-methylcoumarin (Z-LLE-AMC, Enzo Life Sciences), respectively. The mixture, containing 10 μg of total protein in 20 mM Tris (pH 8) and 10% gylcerol, was incubated at 37°C with 20 μM peptide substrates in a final volume of 100 μl. Enzymatic kinetics were monitored in a temperature-controlled microplate fluorimetric reader (FLUOstar Galaxy, BMG labtech). Excitation/emission wavelengths were 350/440 nm. The difference between assays with or without MG-132, a proteasome inhibitor, represented the proteasome-specific activity.

### Relative Quantification of Gene Expression by qPCR

Total RNA was extracted from the plantaris muscle using QIAzol^®^ lysis reagent, TissueLyser II system, and Rneasy minikit (Qiagen France SAS) following the manufacturer’s instructions. Extracted RNA was spectrophotometrically quantified using NanoDrop 2000 (Thermo Fisher Scientific). From 500 ng of extracted RNA, the first-strand cDNA was then synthesized using the Transcriptor First Strand cDNA Synthesis Kit (Roche Diagnostics) with anchored-oligo(dT)18 primer and according to the manufacturer’s instructions. Using a Light Cycler^®^ 480 system (Roche Diagnostics), the reaction was carried out in duplicate for each sample in a 6 μl reaction volume containing 3 μl of SYBR Green Master Mix, 500 nM of the forward and reverse primers each and 3 μl of diluted (1:25) cDNA. The thermal profile for SYBR Green qPCR was 95°C for 8 min, followed by 40 cycles at 95°C for 15 s, 60°C for 15 s and 72°C for 30 s. To exclude PCR products amplified from genomic DNA, primers were designed, when possible, to span one exon-exon junction. Primers sequences used in this study are available on request. The expression of hydroxymethylbilane synthase (Hmbs) and succinate dehydrogenase complex flavoprotein subunit A (Sdha) was used as a reference transcript. At least five animals were used for each experimental point.

### Statistical Analysis

Groups were statistically compared with GraphPad Prism 7 using ordinary two-way analysis of variance. Multiple comparisons were performed to compare means of Basal groups to OVL-treated groups (see Supplementary Table 1). Multiple comparisons were corrected by the Tukey statistical hypothesis testing. Gain between WT and KO groups were statistically compared using unpaired two-tailed *T*-test. If normal distribution (verified using Shapiro-Wilk’s test) and/or equal variance (verified using Bartlett’s test) are not assumed, groups were statistically compared using the test of Wilcoxon-Mann-Whitney. A *p* < 0.05 was considered significant. Values are given as means ± SEM.

## Results

### Reduced Gain of Muscle Performance in Response to Mechanical Overload (OVL) in Desmin Knock-Out Mice

**FIGURE 1:**
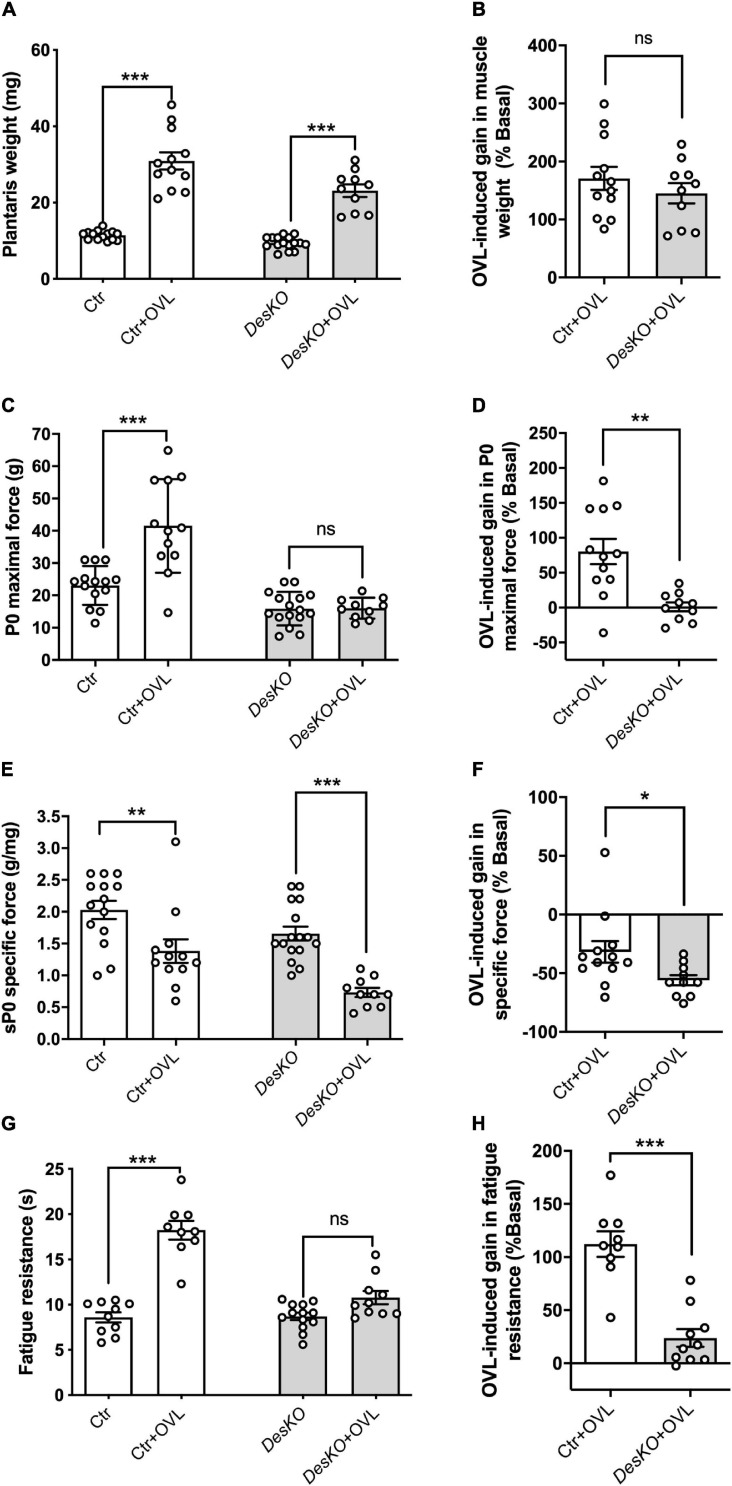
Maximal force of plantaris is not increased in response to OVL in DesKO mice. Muscle weight **(A)**, maximal force (P0, **C**), specific force (sP0, **E**) and fatigue resistance **(G)** were evaluated in both genotypes (Ctr and *Des*KO) in basal condition or after 1 month of OVL. The gain or loss in these parameters was calculated compared to the corresponding basal condition for the Ctr + OVL and *Des*KO + OVL groups **(B,D,F,H)**. Data are given as means ± SEM. *Des*KO, Desmin knock-out mice; Ctr, Control mice; OVL, mechanical overloading. *ns*: non-significant, *p < 0.05, **p < 0.01, ***p < 0.001.

To analyze the role of desmin during adaptation to resistance training, we examined the gains in muscle weight, muscle force generation capacity and fatigue resistance in response to overload (OVL) in *DesKO* and control mice. Plantaris muscles were overloaded by surgical ablation of gastrocnemius and soleus muscles. One month following OVL, muscle weight was markedly upregulated in both genotypes (Figure 1A). The percentage of muscle weight gain compared to the unchallenged muscle was calculated and was not different in both groups (Figure 1B), suggesting that desmin depletion does not prevent muscle mass regulation following resistance training. It should be noted that body weight in *DesKO* mice in basal state was lower compared to control mice (16.80 g ± 0.60 g in *DesKO* vs. 21.24 g ± 0.50 g in Ctr, *p* = 0.0001) and OVL did not modify this difference (18.40 ± 0.15 for *DesKO* + OVL vs. 20.27 ± 0.27 for Ctr + OVL, *p* = 0,0258). *In situ* force production in response to nerve stimulation was analyzed. In contrast to control mice, OVL did not increase absolute maximal force of plantaris muscle in *Des*KO mice (Figures 1C,D). The specific maximal force, which represents the normalization of absolute maximal force by muscle weight, was decreased in response to OVL in both genotypes (Figure 1E) (*p* < 0.05). Notably, this decrease was higher in *Des*KO mice as compared to control mice (Figure 1F) (*p* < 0.05). Fatigue resistance was also analyzed by continuously stimulating plantaris muscle and measuring the time corresponding to a decrease of 50% of initial force. Our results showed that fatigue resistance increased in response to OVL only in control mice (Figures 1G,H) (*p* < 0.05). Taken together, our results indicate that desmin plays an important role in the gain of muscle performance, but not in the gain of muscle weight in response to OVL.

### Muscle Remodeling in Response to Mechanical Overload (OVL) in Desmin Knock-Out Mice

**FIGURE 2:**
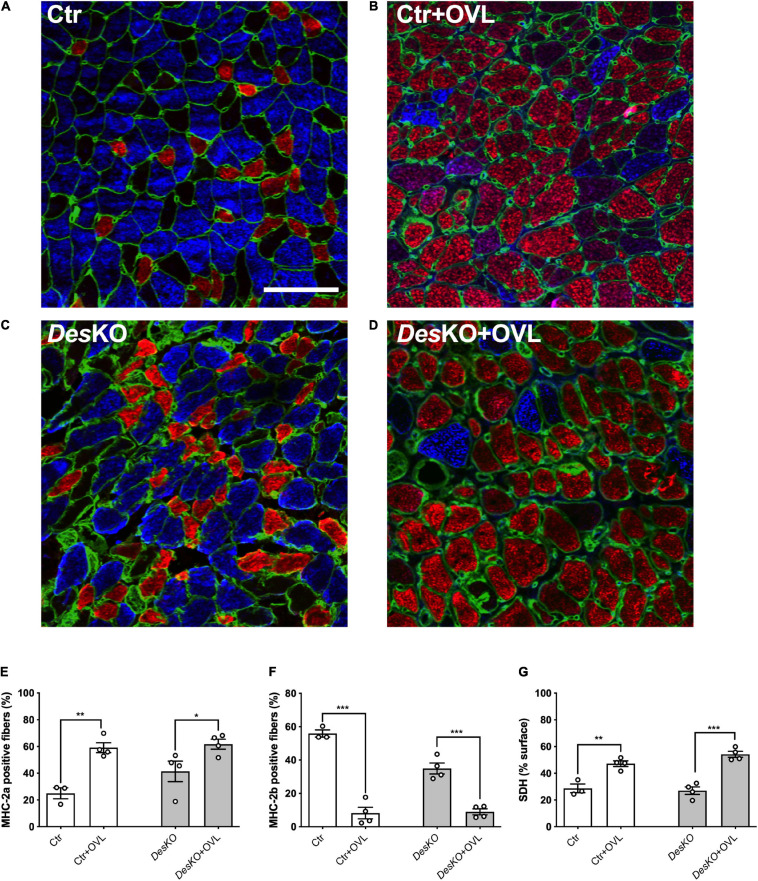
Transition toward oxidative fibers is not impaired in DesKO plantaris in response to OVL. Representative images **(A–D)** of plantaris section immunostained for MHC-2a (red), MHC-2b (blue) and perlecan (green) from both genotype (Ctr and *Des*KO) in basal condition or after 1 month of OVL. Percentage of MHC-2a positive fibers **(E)** or MHC-2b positive fibers **(F)**. Percentage of the surface of muscle section demonstrating succinate dehydrogenase activity **(G)**. Scale bar = 100 μm. Data are given as means ± SEM. *Des*KO, Desmin knock-out mice; Ctr, Control mice; OVL, mechanical overloading; MHC, myosin heavy chain; SDH, succinate dehydrogenase. *ns*: non-significant, *p < 0.05, **p < 0.01, ***p < 0.001.

Since the changes in muscle force generation capacity and fatigue resistance can be related to myosin heavy chain (MHC) composition of muscle fibers, we used immunohistochemistry to compare MHC composition of *Des*KO and control plantaris muscles after 1 month of OVL (Figures 2A–D). The proportion of fibers expressing two major MHC isoforms, MHC-2a (oxidative fiber) and MHC-2b (glycolytic fiber), was analyzed using a custom macro (ImageJ software) to count MHC-positive cells. The proportion of MHC-2a-expressing fibers increased and the proportion of MHC-2b-expressing fibers decreased in response to OVL in both genotypes (Figures 2E,F). Despite the fact that the change in MHC composition of plantaris muscle was found similar between *Des*KO and control mice, the increase rate of the percentage of MHC-2a fiber in response to OVL in *Des*KO mice was lower by more than 2.5-fold compared to control mice (+ 49% ± 9% in *Des*KO vs. + 136% ± 15% in control, *p* = 0.002). However, it should be noted that the percentage of oxidative MHC-2a fibers in *DesKO* mice was already increased before OVL (Figure 2E) (*p* < 0.05), presumably as a consequence of the lack of desmin as previously demonstrated (Agbulut et al., 1996). We also examined the oxidative energetic metabolism of plantaris muscle after 1 month of OVL using succinate dehydrogenase (SDH) staining. Our results indicated higher SDH activity in response to OVL in both genotypes (Figure 2G) (*p* < 0.05). Taken together, our results indicate that the glycolytic to oxidative metabolism transition pattern in response to OVL did not markedly differ between *Des*KO and control mice.

### Myostatin Pathway Mediates Muscle Mass Plasticity in Desmin Knock-Out Mice

**FIGURE 3:**
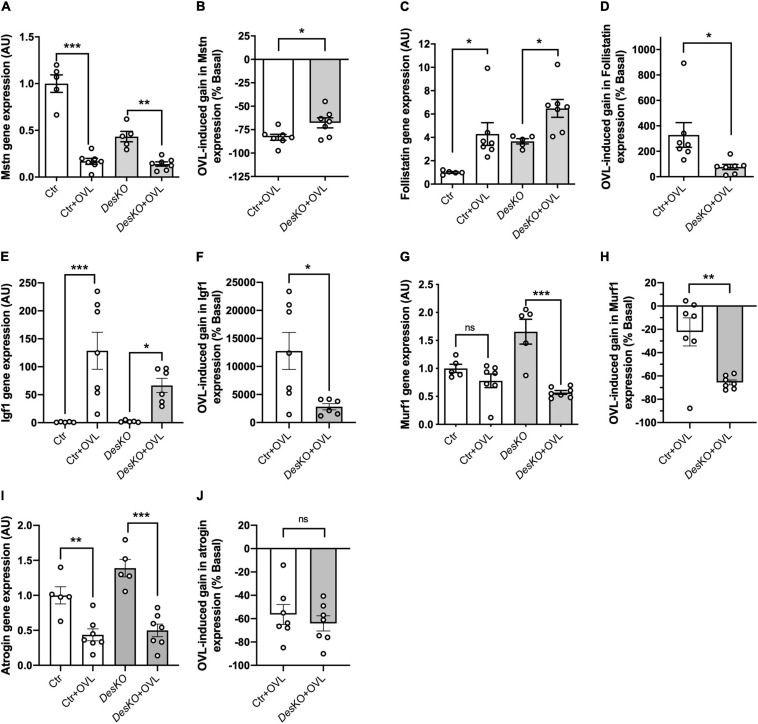
Expression of genes involved in the regulation of muscle mass pathways regulation is modified in DesKO mice after OVL. Gene expression of the hypertrophic markers myostatin **(A,B)**, Follistatin **(C,D)**, Igf1 **(E,F)** and of the anti-hypertrophic markers Murf1 **(G,H)** and Atrogin **(I,J)** were evaluated in both genotype (Ctr and *Des*KO) in basal condition or after 7 days of OVL. The gain or loss of expression of these genes was calculated compared to the corresponding basal condition for the Ctr + OVL and *Des*KO + OVL groups **(B,D,F,H,J)**. Data are given as means ± SEM. *Des*KO, Desmin knock-out mice; Ctr, Control mice; OVL, mechanical overloading. *ns*, non-significant, *p < 0.05, **p < 0.01, ***p < 0.001.

OVL is known to induce muscle hypertrophy and hyperplasia. As several players can be involved in the control of the muscle mass, we examined the mRNA levels of intracellular signaling molecules involved in hypertrophy. Semi-quantitative PCR analysis was performed on muscle samples after 7 days of OVL during the early phase of muscle remodeling (Figures 3A–J). Myostatin (Mstn or Gdf8), an mTOR deactivator, mRNA levels decreased with OVL (Figure 3A), to a lower extent in *DesKO* mice (Figure 3B). In the same line, the transcript of follistatin (Figures 3C,D), an antagonist of myostatin, and insulin growth factor 1 (Igf1) (Figures 3E,F) increased in all genotypes but these increases were lower in *DesKO* compared to control mice muscles. In addition, two other markers MuRF1 and atrogin, which are important markers for skeletal muscle atrophy by promoting protein catabolism and contribute to the decline of muscle mass and strength in sarcopenia, were examined. Our results demonstrated that OVL did not modify MuRF1 expression in control, but strongly reduced it in DesKO mice, suggesting that desmin presence reduces protein degradation upon OVL stimulation (Figures 3G,H). Regarding atrogin expression, no stricking difference was observed after OVL between the two genotypes (Figures 3I,J). Taken together, our data suggest that conventional muscle mass regulation pathways are activated in *DesKO* mice and contribute to the plasticity of muscle mass in OVL-elicited remodeling.

**FIGURE 4:**
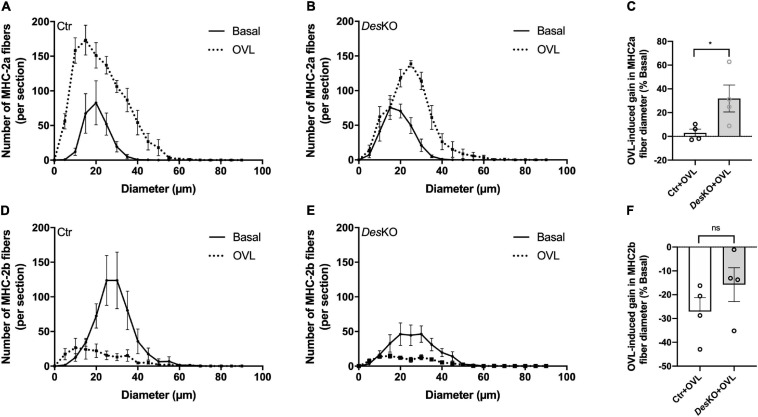
Size distribution of plantaris muscle fibers is modified in DesKO mice after OVL. Size distribution of plantaris muscle fibers expressing only MHC-2a **(A,B)** or MHC-2b **(D,E)** for both genotypes (Ctr and *Des*KO) in basal condition (black line) or after 1 month of OVL (black dotted line). The gain or loss in these parameters was calculated compared to the corresponding basal condition for the Ctr + OVL and *Des*KO + OVL groups **(C,F)**. Data are given as means ± SEM. For the size distribution, *n* = 4 for all conditions (*n* = 3 for Ctr + Basal group). *Des*KO, Desmin knock-out mice; Ctr, Control mice; OVL, mechanical overloading; MHC, myosin heavy chain. *ns*, non-significant, *p < 0.05.

To complete these results, we analyzed the number and the size distribution of the MHC-2a and the MHC-2b muscle fibers on cross sections of plantaris muscle in basal condition and 1 month after OVL. As demonstrated in Figure 4, the number of MHC-2a fibers was increased in both genotypes. Interstingly, in control mice the size distribution of MHC-2a fibers was not modified by OVL. On the other hand, in *DesKO* mice, the size distribution was slightly shifted toward the higher values indicating an asymmetrical increase in the number of MHC-2a fibers with high diameter (Figures 4A,B). Thus, the mean diameter of MHC-2a fibers was increased in response to OVL in *DesKO* mice but not in control mice (+ 31.91% ± 11.32% in *DesKO* vs. + 2.93% ± 3.18% in control, *p* = 0.048) (Figure 4C). As expected, after 1 month of OVL, the number of MHC-2b fibers was decreased in both genotypes (Figures 4D,E). However, it seems that the decrease in the mean size of MHC-2b fibers in response to OVL was more important in control mice compared to *DesKO* mice although the difference did not reach statistical significance (–15.79% ± 7.09% in *DesKO* vs. –27.11% ± 5.87% in control, *p* = 0.264) (Figure 4F).

### Mechanisms Responsible for the Reduced Gain of Performance in Desmin Knock-Out Mice

**FIGURE 5:**
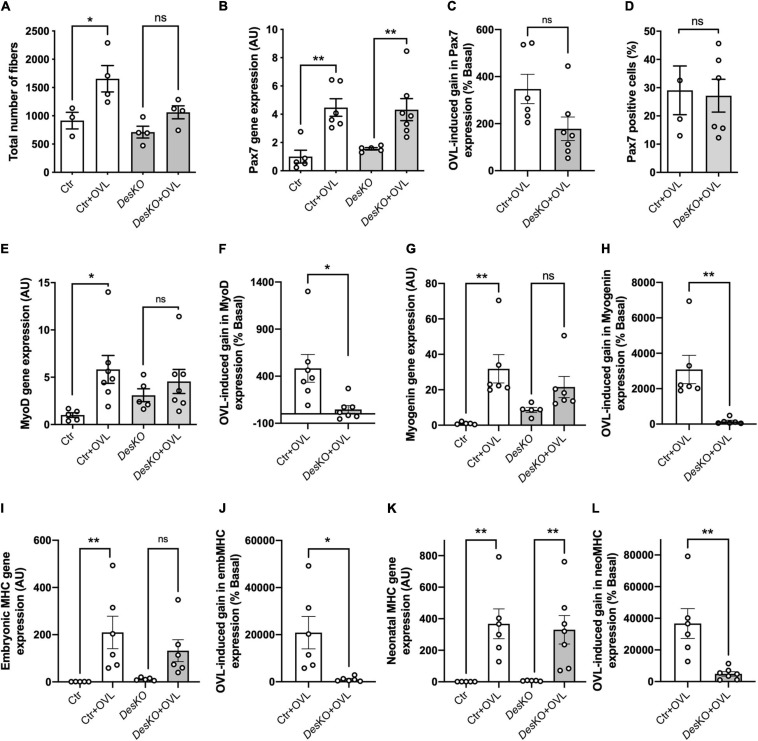
The myogenic program is disturbed in DesKO after OVL. Total number of fibers of plantaris muscle were evaluated after 1 month of OVL **(A)**. Gene expression analysis for Pax7 **(B,C)**, MyoD **(E,F)**, Myogenin **(G,H)**, embryonic MHC **(I,J)** and neonatal MHC **(K,L)** were evaluated in both genotype (Ctr and *Des*KO) in basal condition or after 7 days of OVL. The gain or loss of expression of these genes was calculated compared to the corresponding basal condition for the Ctr + OVL and *Des*KO + OVL groups **(C,F,H,J,L)**. Moreover, Pax7 positive cells were counted after 7 days of OVL **(D)**. Data are given as means ± SEM. *Des*KO, Desmin knock-out mice; Ctr, Control mice; OVL, mechanical overloading; MHC, myosin heavy chain. *ns*: non-significant, *p < 0.05, **p < 0.01.

To determine the mechanisms involved in the deficit of muscle function observed in *DesKO* muscles challenged with OVL, myofiber number were quantified in plantaris muscles. Myofiber number was increased by OVL in control muscle, but not in *DesKO* (Figure 5A). A defect in myogenesis affecting *DesKO* muscles could lead to less myofiber formation following OVL, and could participate in the observed deficit in muscle function. Thus, Pax7 transcripts were quantified (Figure 5B). Pax7 expression increased in response to OVL both in control and *DesKO* mice while there was no statistically significant difference observed between the two genotypes (Figures 5B,C). To support this result, we also quantified Pax7 positive cells in *DesKO* and control mice after OVL (Figure 5D). Our results show no difference in the ratio of Pax7 positive cells between control and *Des*KO mice in response to OVL, suggesting that MuSC are not depleted in absence of desmin. However, we found that the induction of MyoD expression due to OVL was repressed in *DesKO* muscle, suggesting that MuSC activation is repressed (Figures 5E,F). Consistent with this observation, myogenin, neonatal and embryonic MHC were less induced by OVL in *DesKO* mice compared to control mice muscles (Figures 5G–L). Unchanged Pax7 expression associated with decreased markers for regeneration in response to OVL in *DesKO* compared to control muscles, pleads for a reduction in the myogenic program affecting *DesKO* MuSCs in response to OVL. In order to explore the consequences of this reduced myogenic program on the capacity of *DesKO* muscle to repair muscle damage due to the mechanical overloading, we evaluated the extent of fibrosis and inflammatory response in *DesKO* and control mice. Fibrosis was first assessed by Sirius red staining after 1 month of OVL (Supplementary Figure 1A). No difference was found between the two genotypes. Moreover, qPCR analyses were performed on muscle samples 7 days after OVL during the early phase of muscle remodeling (Supplementary Figure 1B). The mRNA levels of the fibrosis and inflammation markers Il1b, Tgfβ1, Col3a1, Col1a1 and Timp1 increased strongly in response to OVL in a similar manner in both genotypes (*p* < 0.05). We also studied protein kinase PKA since it contributes to muscle regeneration (Stewart et al., 2011). We found that the levels of the phosphorylated forms of PKA regulatory subunit IIα (PKA RIIα) protein increased in control mice in response to OVL but not in *Des*KO mice (Supplementary Figures 1C,D). Taken together, despite minor modifications observed in *Des*KO mice compared to control, the absence of desmin does not impair muscle fiber regeneration.

**FIGURE 6:**
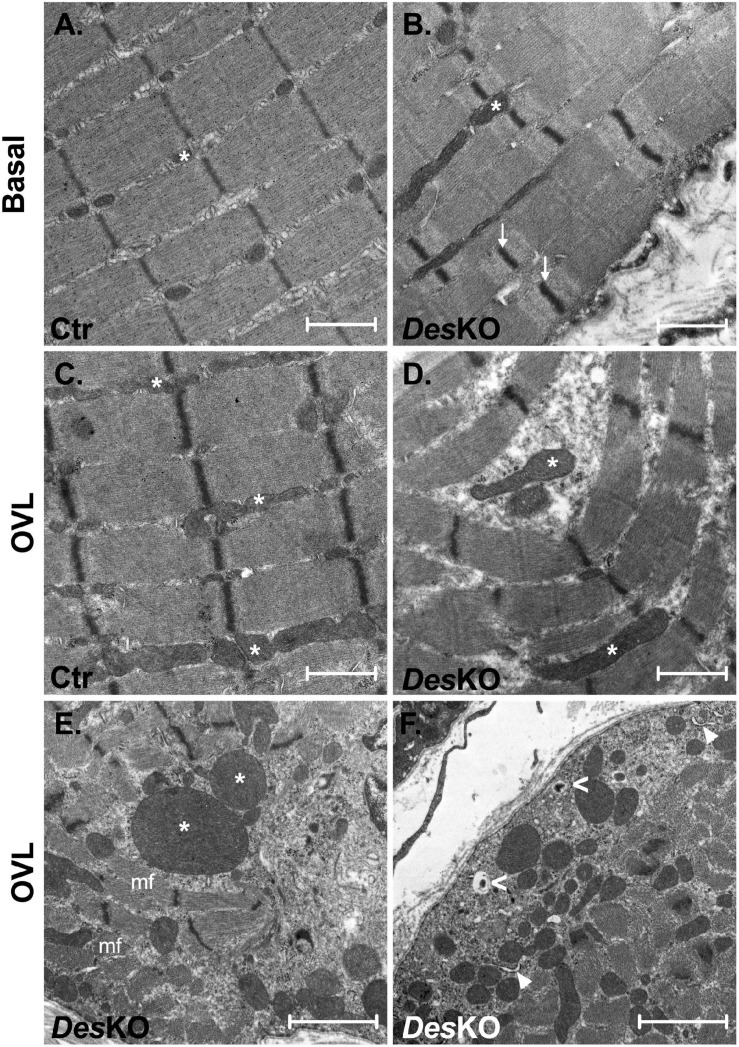
Myofibrils organization of plantaris is highly disturbed in DesKO after OVL. Transmission electron microscopy images of ultrathin sections of plantaris of both genotype (Ctr and *Des*KO) in basal condition or after 1 month of OVL. Asterisks (*) indicate mitochondria; white arrows indicate Z-lines; mf indicates myofibrils; white arrowheads indicate autophagosome; white empty arrowheads indicate autolysosome. *Des*KO, Desmin knock-out mice; Ctr, Control mice; OVL, mechanical overloading. **(A–D)** Scale bar = 1 μm. **(E,F)** Scale bar = 2 μm.

**FIGURE 7:**
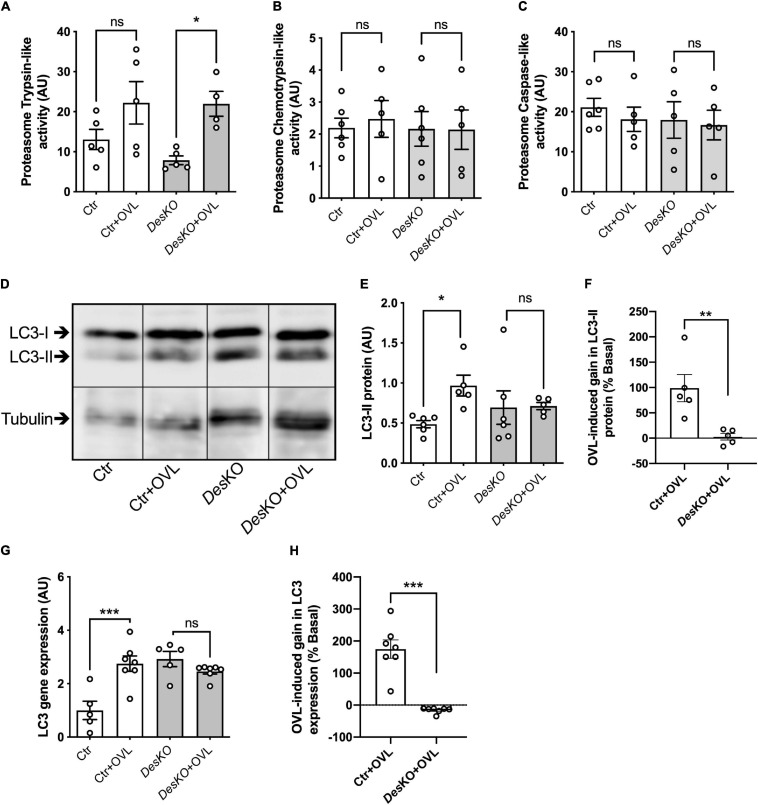
Proteostasis pathways are disturbed in DesKO after OVL. The trypsin-like activity **(A)**, chymotrypsin-like activity **(B)** and caspase-like activity **(C)** of proteasome were measured in both genotype (Ctr and *Des*KO) in basal condition or after 1 month of OVL. LC3-II protein **(D–F)** and LC3 gene expression **(G,H)** were quantified in both genotype (Ctr and *Des*KO) in basal condition or after 1 month of OVL. Data are given as means ± SEM. *Des*KO, Desmin knock-out mice; Ctr, Control mice; OVL, mechanical overloading. *ns*: non-significant, *p < 0.05, **p < 0.01, ***p < 0.001.

To better understand the morphological perturbations affecting *DesKO* muscles, we also examined the ultrastructure of the plantaris muscle fibers using transmission electron microscopy (Figure 6). In comparison to control muscles, *Des*KO myofibers present increased number of mitochondria and irregularities in the organization of the myofibrils, with misalignment of Z-lines (Figures 6A,B). One month after OVL, control mice present only minor modifications, i.e., increase of the number and the size of mitochondria (Figure 6C, see asterisk). In contrast, *Des*KO mice presented serious muscle damages as indicated by the loss of sarcomere integrity, alignment and orientation, and abnormalities in size, number and distribution of mitochondria (Figures 6D,E). Moreover, mitochondria appeared swollen and accumulated in the muscle fibers. As presented in Figure 6F, an accumulation of autophagosomes was spotted under the sarcolemma (white arrowheads) and lysosomes (white empty arrowheads) in *Des*KO mice after 1 month of OVL. These observations could indicate a perturbation of autophagy. Indeed, an impairment of proteolysis mechanisms leading to the accumulation of unfunctional proteins, such as proteins contributing to muscle contraction, could participate in the reduced gain of performance in *Des*KO mice. To address this possibility, we explored two main proteolysis pathways, the ubiquitin-proteasome system and the autophagy, in *Des*KO and control mice and in response to OVL. Regarding the ubiquitin-proteasome system, we measured chymotrypsin-like, trypsin-like and caspase-like activities of the proteasome 20S catalytic core using the fluorogenic substrates Suc-LLVY-AMC in *Des*KO and control muscle homogenates after 1 month of OVL. Chemotrypsin-like and caspase-like activities show no difference between *Des*KO and control mice (Figures 7B,C). However, the proteasome trypsin-like activity was increased in response to OVL in *Des*KO mice but not in control mice (Figure 7A) (*p* < 0.05). Concerning autophagy, we examined LC3-II protein level by western-blotting in *Des*KO and control plantaris muscle 1-month after OVL (Figure 7D). Our results show that both mRNA and protein levels of LC3-II did not change in *Des*KO mice, while they increased in control mice, in response to OVL (Figures 7D–H) (*p* < 0.05). It should be noted that the mRNA levels of LC3 were higher in DesKO mice at the baseline compared to the control. Together, these results underline alteration of proteolysis pathways in *Des*KO mice in response to OVL.

## Discussion

Skeletal muscle responds to resistance training by activating adaptation mechanisms at the cellular level, which result in muscle fiber growth and regeneration, increase in fatigue resistance, and gain of force (Joanne et al., 2012). In this study, we used the desmin-deficient (*Des*KO) female mouse to address the role of desmin in the response of skeletal muscle to mechanical overload (OVL), a well-studied experimental model which mimics resistance training. We found that, in response to OVL, gain in performance is not fully achieved in the absence of desmin, despite notable muscle remodeling and in relation with impaired proteolysis.

### Muscle Remodeling Is Affected by Desmin Depletion in Response to OVL

One month after surgical ablation of gastrocnemius and soleus muscles, the plantaris muscle of both *Des*KO and control mice responded by an important increase in weight (Figure 1), suggesting resistance training-induced muscle growth (Joanne et al., 2012). Both genotypes also displayed a fiber type switch toward a more oxidative phenotype, which is consistent with increased fatigue resistance. In particular, MHC2b-positive, glycolytic fibers are partially replaced by MHC2a-positive and more oxidative fibers. Also, mitochondrial activity, as detected by SDH staining, appeared increased by twofold (Figure 2). Gene expression levels of mTOR deactivator myostatin decreased whereas follistatin, an antagonist of myostatin, and the positive regulator of muscle growth Igf1 transcripts increased in both genotypes, also suggesting that the hypertrophy process is activated (Figure 3). Igf1 is also implicated in muscle regeneration by promoting both proliferation and differentiation of MuSCs (Jang et al., 2011). However, desmin-deficiency toned down both OVL-elicited myostatin reduction and stimulated follistatin expression (Figures 3A–D). This suggests that the regulation of muscle mass is not affected by desmin deficiency. Overloaded *Des*KO and control muscles showed increase in gene expression of Pax7, embryonic MHC and neonatal MHC (Figure 5). These results suggest that hyperplasia and regeneration also occur in both genotypes. Interestingly, OVL does not increase myofiber number in *DesKO* muscles, although it does in control mice (Figure 5A). While Pax7-positive cells, and OVL-induced gain of Pax7 expression was identical in control and *DesKO* muscles. However, OVL-induced upregulation of MyoD, Myogenin, neonatal, and embryonic MHC were dampened in *DesKO* muscles (Figure 5). Our data suggest that the myogenic program triggered by OVL stimulation is only partially supported in absence of desmin. We also examined differences in phosphorylation of PKARIIα which leads to an increase in protein synthesis, in favor of hypertrophy and muscle growth. Although the levels of phosphorylated PKARIIα increased only in the control muscle after OVL, these levels were already elevated in the *Des*KO in the basal condition. A possible explanation may be related to another intermediate filament, synemin. Synemin is involved in the control of hypertrophy by modulating the subcellular localization of PKA. We have previously reported higher phosphorylated levels of PKA and increased hypertrophy in the synemin-deficient skeletal muscle as compared to control mice and in response to OVL (Li et al., 2014). Formation of synemin filaments in muscle requires copolymerization with desmin. Consequently, synemin appears unstable and delocalized in *Des*KO muscle fibers (Carlsson et al., 2000), providing one possible explanation for the higher levels of phosphorylated PKARII in *Des*KO under basal conditions. Taken together, these results suggested that the process of muscle fiber remodeling was activated. Muscle fiber type switch as well as hypertrophy and regeneration appear to occur in both genotypes as a response to OVL. These processes are not impaired by the lack of desmin; however, our data suggest that muscle remodeling is moderated in response to OVL.

### Impaired Gain in Performance in the Absence of Desmin

Interestingly, here we show that, despite muscle hypertrophy, maximal force production is not improved in *Des*KO plantaris muscle in response to OVL. It was known that muscle hypertrophy does not necessarily increase maximal force production as shown in the case of myostatin inhibition (Stantzou et al., 2017). Increased fibrosis and inflammation as compared to control could be a possible partial explanation for the fact that maximal force was not proportional to muscle weight in *Des*KO (Costamagna et al., 2015). However, although gene expression of proteins involved in inflammation and fibrosis greatly increased after 7 days of OVL, this increase was similar for both genotypes and no statistically significant increase in fibrosis was measured *in situ* on muscle sections, at least after 1 month of OVL (Supplementary Figure 1). Efficient muscle fiber contraction can be impaired by structural disorganization of sarcomeres, and in particular by misalignment, disintegration, and loss of myofibrils (Li et al., 1997). Electron microscopy analysis (Figure 6) showed that OVL did not destabilize the contractile apparatus in control muscle fibers. The main effect was the increased number of mitochondria, as expected by the switch toward a more oxidative metabolism. On the contrary, OVL had major structural consequences in *Des*KO muscle. Under basal conditions, *Des*KO muscle fibers displayed intermyofibrillar accumulation of mitochondria as well as misaligned Z-lines. However, the contractile apparatus was not disintegrated. After OVL, *Des*KO muscle fibers appeared damaged, with abnormally accumulated and often swollen mitochondria as well as misaligned, disintegrated and disoriented myofibrils, as shown by the coexistence of longitudinal and cross-sectioned myofibrils within the same muscle fiber (Figure 6E). A similar cellular phenotype has been previously described for *Des*KO muscle fibers in basal condition (Milner et al., 1996; Li et al., 1997), although only for slow-twitch muscles and in older mice (≥5 months-old). Therefore, we conclude that in the absence of desmin, muscle fiber growth cannot compensate the increased structural damage of the contractile apparatus, and is not sufficient to improve force production.

It is known that accumulation of damaged mitochondria leads to increased reactive oxygen species generation, decreased ATP production, cellular dysfunction, and finally cell death (Bloemberg and Quadrilatero, 2019). Intense muscle effort under OVL conditions results in production of damaged proteins and organelles which need to be effectively cleared. We examined the two major proteolytic pathways, the ubiquitin-proteasome system and autophagy, in *Des*KO and control muscles. Proteasomal activity was reported in OVL experiments (Baehr et al., 2014), and the role of autophagy in muscle adaptation to exercise has been extensively studied and established (Lira et al., 2013; Luo et al., 2013). In our experiments, in response to OVL, the proteasome activity appeared partially activated (trypsin-like activity), whereas the levels of LC3-II, the activated (lipidated) form of autophagy marker LC3 protein, did not increase in *Des*KO muscle fibers. On the contrary, both LC3 gene expression and LC3-II protein levels increased in control muscle fibers after OVL. Interestingly, LC3 gene expression was already increased in *Des*KO muscle fibers under basal conditions. Moreover, electron microscopy reveals the presence of autophagosomes and autolysosomes in the cytoplasm of *Des*KO muscle fibers after OVL (Figure 6F). One possible explanation could be that although the process is activated, the autophagy machinery (autophagosome production, fusion to lysosomes and clearance) in *Des*KO is already at maximum capacity in basal state because of high production of damaged cell material due to the lack of desmin, and is getting inefficient under OVL conditions. This hypothesis is supported by the accumulation of damaged mitochondria under OVL conditions (Figure 6). Interestingly, it has been previously shown that blockage of autophagy in muscle-specific *Atg7*-null mice resulted in impairment of force transmission and in accumulation of dysfunctional mitochondria (Masiero et al., 2009). We therefore propose that, after OVL and in the absence of desmin, the repairing activity of autophagy could be disturbed and this may at least partially explain the accumulation of damaged mitochondria and dysfunctional contractile proteins which leads to compromised integrity of muscle and reduced production of specific maximal force, as was shown in other cases (Wohlgemuth et al., 2010). Further studies are required to elucidate the role of autophagy in our model system by generating functional data of autophagy inhibition/promotion *in vivo*.

## Conclusion

In conclusion, here we show that during muscle remodeling in response to OVL, muscle growth and increase in force production can be dissociated in *Des*KO mice. It has been proposed that, during resistance exercise, force transmission at the myofibril level must be supported by the cytoskeleton, as suggested by increased expression of desmin (Parcell et al., 2009). We propose that mechanical OVL increases the negative impact of the lack of desmin on myofibril organization and mitochondria. Furthermore, our results suggest that under these conditions, the repairing activity of autophagy is impaired. Consequently, force generation is not improved despite muscle growth, suggesting that desmin is required for a complete response to resistance training in skeletal muscle.

## Data Availability Statement

The original contributions presented in the study are included in the article/Supplementary Material, further inquiries can be directed to the corresponding author/s.

## Ethics Statement

All animal studies were approved by our institutional Ethics Committee (Charles Darwin, project number: 01362.02) and conducted according to the French and European laws, directives, and regulations on animal care (European Commission Directive 86/609/EEC).

## Author Contributions

PJ, MB, OA, AF, and EK contributed to the data collection, the statistical analysis, the interpretation, and the manuscript writing. PJ, YH, MB, and JG-L carried out the histological and immunostainig and molecular analysis. AL, YH, M-TD, AP, and ZL carried out the western blot experiments and proteasome activity measurement and analysis. GT and OA carried out the electron microscopy experiments. AF carried out the muscle force measurements. OA and AF designed and supervised the research. All authors read and approved the final manuscript.

## Conflict of Interest

The authors declare that the research was conducted in the absence of any commercial or financial relationships that could be construed as a potential conflict of interest. The reviewer VM declared a past co- authorship with the authors ZL, OA to the handling editor.

## Supplementary Material

Supplementary Figure Fibrosis and inflammation are not impaired in DesK0 after OVL. Quantification of fibrosis **(A)** and relative gene expression of inflammation and fibrosis markers **(B)**. Quantification of the phosphorylation of PKA (pPKA, **C,D**). Parameters were measured in both genotypes (Ctr and *Des*KO) in basal condition or after one month of OVL. For gene expression analysis,*n* = 5 for Ctr groups and *n* = 7 for OVL groups. Data are given as means±SEM. *Des*KO, Desmin knock-outmice; Ctr, Control mice; OVL, mechanical overloading. *ns*: non significant, **p* < 0.05, ***p* < 0.01.

Supplementary Table Number of samples for each evaluated parameter and *p*-values following two-way ANOVA analysis. DesKO, Desmin knock-out mice; Ctr, Control mice; OVL, mechanical overloading; ns, non-significant.

## Abbreviations

*Des*KO: desmin knock-out mice
GAPDH: glyceraldehyde 3-phosphate dehydrogenase
Hmbs: hydroxymethylbilane synthase
Igf1: insulin growth factor 1
i.p.: intraperitoneal
MHC: myosin heavy chain
Mstn: Myostatin
MuSCs: Muscle stem cells
OVL: mechanical overloading
Perlecan: heparan sulfate proteoglycan
PKARIIα: protein kinase A regulatory subunit IIα
P0: maximal force
SDH: succinate deshydrogenease
Sdha: succinate dehydrogenase complex flavoprotein subunit A
sP0: specific maximal force.
