# Supplementary material for: Absence of Desmin Results in Impaired Adaptive Response to Mechanical Overloading of Skeletal Muscle
Source: Front Cell Dev Biol. 2021 Jul 15;9:662133. doi: 10.3389/fcell.2021.662133 (PMC8320001; doi:10.3389/fcell.2021.662133)
Supplement: Supplementary Figure — Fibrosis and inflammation are not impaired in DesK0 after OVL. Quantification of fibrosis (A) and relative gene expression of inflammation and fibrosis markers (B). Quantification of the phosphorylation of PKA (pPKA, C,D). Parameters were measured in both genotypes (Ctr and DesKO) in basal condition or after one month of OVL. For gene expression analysis,n = 5 for Ctr groups and n = 7 for OVL groups. Data are given as means±SEM. DesKO, Desmin knock-outmice; Ctr, Control mice; OVL, mechanical overloading. ns: non significant, *p < 0.05, **p < 0.01. [file Data_Sheet_1.pdf]

Supplementary Figures

Absence of desmin results in impaired adaptive response to mechanical overloading of skeletal muscle

Pierre Joanne[1], Yeranuhi Hovhannisyan[1], Maximilien Bencze[2], Marie-Thérèse Daher[1], Ara Parlakian[1], Geraldine Toutirais[3], Jacqueline Gao-Li[1], Alain Lilienbaum[4], Zhenlin Li[1], Ekaterini Kordeli[1], Arnaud Ferry[5,6], Onnik Agbulut[1*]

[1] Sorbonne Université, Institut de Biologie Paris-Seine (IBPS), CNRS UMR 8256, Inserm ERL U1164, Biological Adaptation and Ageing, 75005, Paris-France.

[2] U955-IMRB, Team 10, Biology of the Neuromuscular System, Inserm, UPEC, ENVA, EFS, Créteil 94000, France.

[3] Muséum National d'Histoire Naturelle (MNHN), Unité Molécules de Communication et Adaptation des Micro-organismes (MCAM), CNRS UMR 7245, Plateau technique de Microscopie Electronique (PtME), 75005, Paris-France.

[4] Université de Paris, Unité de Biologie Fonctionnelle et Adaptative, CNRS UMR 8251, 75013, Paris-France.

[5] Sorbonne Université, Institut de Myologie, INSERM U974, Centre de recherche en myologie, 75013, Paris-France.

[6] Université de Paris, 75006, Paris-France.

Corresponding author :

Prof Onnik Agbulut, Institut de Biologie Paris-Seine, UMR CNRS 8256, 7, quai St Bernard (case 256), 75005 Paris-France. Email : onnik.agbulut@sorbonne-universite.fr

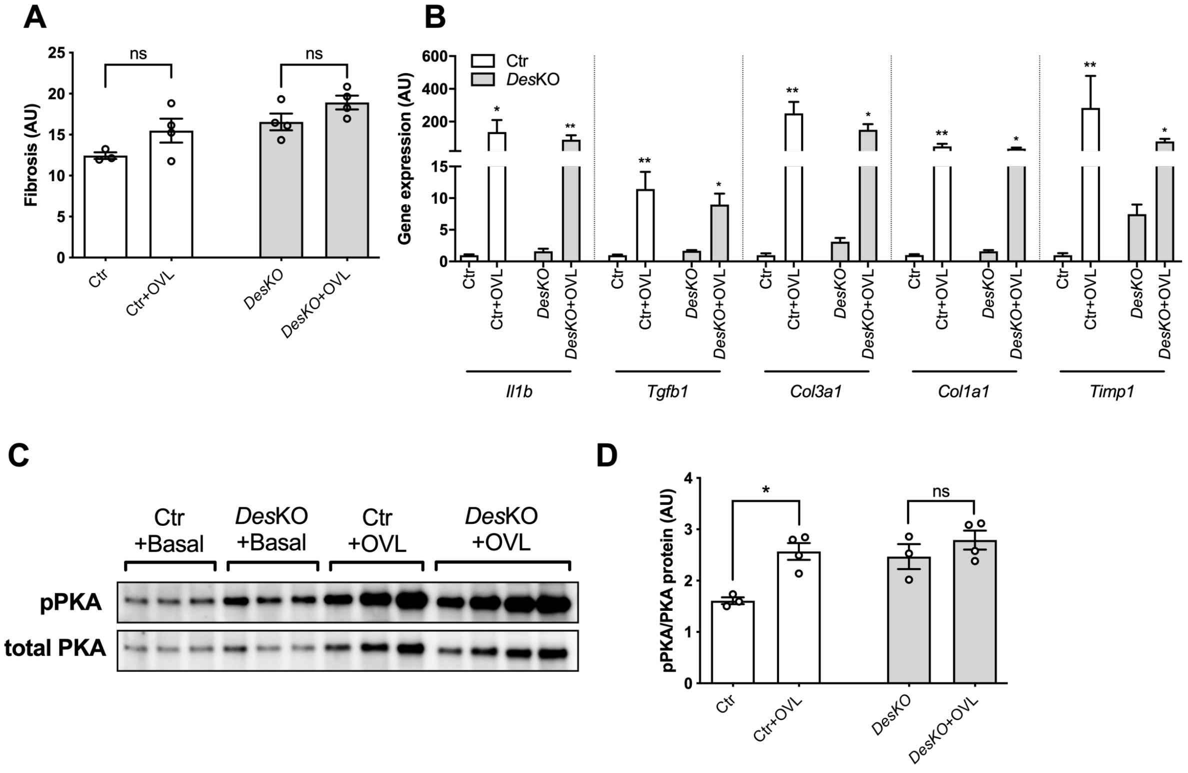

Supplemental Figure 1. Fibrosis and inflammation are not impaired in DesK0 after OVL. Quantification of fibrosis (A) and relative gene expression of inflammation and fibrosis markers (B). Quantification of the phosphorylation of PKA (pPKA, C-D). Parameters were measured in both genotypes (Ctr and *Des*KO) in basal condition or after one month of OVL. For gene expression analysis, n=5 for Ctr groups and n=7 for OVL groups. Data are given as means±SEM. *Des*KO, Desmin knock-out mice; Ctr, Control mice; OVL, mechanical overloading. ns: non significant, *: *p-value*<0.05, **: *p-value*<0.01.
