# Supplementary material for: Absence of Desmin Results in Impaired Adaptive Response to Mechanical Overloading of Skeletal Muscle
Source: Front Cell Dev Biol. 2021 Jul 15;9:662133. doi: 10.3389/fcell.2021.662133 (PMC8320001; doi:10.3389/fcell.2021.662133)
Supplement: Supplementary Table — Number of samples for each evaluated parameter and p-values following two-way ANOVA analysis. DesKO, Desmin knock-out mice; Ctr, Control mice; OVL, mechanical overloading; ns, non-significant. [file Data_Sheet_2.PDF]

Table S1. Number of samples for each evaluated parameter and *p* values following two-way ANOVA analysis.

Evaluated Parameters	Corresponding Figures	Duration of mechanical overloading	Number of samples				Two-way ANOVA		Multiple comparasions (Tukey)			
			Ctr	Ctr+OVL	DesKO	DesKO+OVL	OVL	Genotype	Ctr vs Ctr+OVL	KO vs KO-OVL	Ctr vs KO	Ctr+OVL vs KO-OVL
Plantaris weight (mg)	Figure 1	1 month	14	12	16	10	p = < 0.001	p = < 0.001	p = < 0.001	p = < 0.001	p = *0.63*	p = 0.001
P0 maximal force (g)	Figure 1	1 month	14	12	16	10	p = < 0.001	p = < 0.001	p = < 0.001	p = > 0.999	p = 0.095	p = < 0.001
sP0 specific force (g/mg)	Figure 1	1 month	14	12	16	10	p = < 0.001	p = < 0.001	p = 0.009	p = < 0.001	p = 0.181	p = 0.017
Fatigue resistance (s)	Figure 1	1 month	10	9	13	10	p = < 0.001	p = < 0.001	p = < 0.001	p = 0.159	p = > 0.999	p = < 0.001
MHC-2a positive fibers (%)	Figure 2	1 month	3	4	4	4	p = < 0.001	p = 0.100	p = 0.005	p = 0.049	p = 0.211	p = 0.982
MHC-2b positive fibers (%)	Figure 2	1 month	3	4	4	4	p = < 0.001	p = 0.005	p = < 0.001	p = < 0.001	p = 0.002	p = 0.997
SDH (% surface)	Figure 2	1 month	3	4	4	4	p = < 0.001	p = 0.325	p = 0.002	p = < 0.001	p = 0.969	p = 0.246
Mstn gene expression (AU)	Figure 3	7 days	5	7	5	7	p = < 0.001	p = < 0.001	p = < 0.001	p = 0.003	p = < 0.001	p = 0.970
Follistatin expression (AU)	Figure 3	7 days	5	7	5	7	p = < 0.001	p = 0.004	p = 0.011	p = 0.029	p = 0.123	p = 0.138
Igf1 gene expression (AU)	Figure 3	7 days	5	7	5	7	p = < 0.001	p = 0.178	p = 0.002	p = 0.049	p = > 0.999	p = 0.170
Murf1 gene expression (AU)	Figure 3	7 days	5	7	5	7	p = < 0.001	p = 0.082	p = 0.367	p = < 0.001	p = 0.009	p = 0.367
Atrogin gene expression (AU)	Figure 3	7 days	5	7	5	7	p = < 0.001	p = 0.042	p = 0.004	p = < 0.001	p = 0.047	p = 0.641
Total number of fibers	Figure 4	1 month	3	4	4	4	p = 0.006	p = 0.032	p = 0.042	p = 0.424	p = 0.828	p = 0.084
Pax7 gene expression (AU)	Figure 4	7 days	5	7	5	7	p = < 0.001	p = 0.749	p = 0.005	p = 0.022	p = 0.933	p = 0.998
MyoD gene expression (AU)	Figure 4	7 days	5	7	5	7	p = 0.017	p = 0.737	p = 0.047	p = 0.827	p = 0.673	p = 0.847
Myogenin gene expression (AU)	Figure 4	7 days	5	7	5	7	p = < 0.001	p = 0.814	p = 0.005	p = 0.380	p = 0.789	p = 0.531
Embryonic MHC gene expression (AU)	Figure 4	7 days	5	7	5	7	p = 0.002	p = 0.477	p = 0.023	p = 0.282	p = 0.999	p = 0.605
Neonatal MHC gene expression (AU)	Figure 4	7 days	5	7	5	7	p = < 0.001	p = 0.834	p = 0.015	p = 0.029	p = > 0.999	p = 0.981
Proteasome Trypsin-like activity (AU)	Figure 6	1 month	5	5	5	4	p = 0.004	p = 0.441	p = 0.251	p = 0.046	p = 0.695	p = >0.999
LC3-II protein (AU)	Figure 6	1 month	6	5	6	5	p = 0.078	p = 0.859	p = 0.004	p = 0.994	p = 0.665	p = 0.579
LC3 gene expression (AU)	Figure 6	7 days	5	7	5	7	p = 0.022	p = 0.005	p = < 0.001	p = 0.522	p = < 0.001	p = 0.632
Fibrosis	Figure S2	1 month	3	4	4	4	p = 0.030	p = 0.005	p = 0.276	p = 0.413	p = 0.100	p = 0.149
Il1b gene expression (AU)	Figure S2	7 days	5	7	5	7	p = < 0.001	p = 0.421	p = 0.033	p = 0.005	p = 0.979	p = 0.432
Tgfb1 gene expression (AU)	Figure S2	7 days	5	7	5	7	p = < 0.001	p = 0.629	p = 0.004	p = 0.039	p = 0.994	p = 0.733
Col3a1 gene expression (AU)	Figure S2	7 days	5	7	5	7	p = < 0.001	p = 0.307	p = 0.006	p = 0.048	p = > 0.999	p = 0.370
Col1a1 gene expression (AU)	Figure S2	7 days	5	7	5	7	p = < 0.001	p = 0.501	p = 0.006	p = 0.046	p = > 0.999	p = 0.688
Timp1 gene expression (AU)	Figure S2	7 days	5	7	5	7	p = < 0.001	p = 0.912	p = 0.005	p = 0.017	p = > 0.999	p = 0.998
pPKA/PKA protein (AU)	Figure S2	1 month	3	4	3	4	p = 0.005	p = 0.013	p = 0.016	p = 0.605	p = 0.043	p = 0.785
Proteasome Chemotrypsin-like activity (AU)	Figure S3	1 month	6	5	6	5	p = 0.806	p = 0.726	p = 0.979	p = 0.999	p = 0.999	p = 0.970
Proteasome Caspase-like activity (AU)	Figure S3	1 month	5	5	5	4	p = 0.539	p = 0.508	p = 0.993	p = 0.958	p = 0.9076	p = 0.991

DesKO, Desmin knock-out mice; Ctr, Control mice; OVL, mechanical overloading; ns, non significant.
